# Iron Oxide Nanoparticles in Cancer Treatment: Cell Responses and the Potency to Improve Radiosensitivity

**DOI:** 10.3390/pharmaceutics15102406

**Published:** 2023-09-30

**Authors:** Maria V. Shestovskaya, Anna L. Luss, Olga A. Bezborodova, Valentin V. Makarov, Anton A. Keskinov

**Affiliations:** 1 Federal State Budgetary Institution “Centre for Strategic Planning and Management of Biomedical Health Risks” of the Federal Medical Biological Agency, Schukinskaya st. 5/1, Moscow 119435, Russia; aluss@cspfmba.ru (A.L.L.);; 2 The Department of Technology of Chemical, Pharmaceutical and Cosmetic Products Mendeleev of University of Chemical Technology of Russia, Miusskaya sq. 9, Moscow 125047, Russia; 3 P. Hertsen Moscow Oncology Research Institute of the National Medical Research Radiological Centre, Ministry of Health of the Russian Federation, 2nd Botkinskiy p. 3, Moscow 125284, Russia; olgabezborodova@yandex.ru

**Keywords:** radiosensitization, iron oxide nanoparticles, iron reactivity, cancer treatment, ionizing radiation

## Abstract

The main concept of radiosensitization is making the tumor tissue more responsive to ionizing radiation, which leads to an increase in the potency of radiation therapy and allows for decreasing radiation dose and the concomitant side effects. Radiosensitization by metal oxide nanoparticles is widely discussed, but the range of mechanisms studied is not sufficiently codified and often does not reflect the ability of nanocarriers to have a specific impact on cells. This review is focused on the magnetic iron oxide nanoparticles while they occupied a special niche among the prospective radiosensitizers due to unique physicochemical characteristics and reactivity. We collected data about the possible molecular mechanisms underlying the radiosensitizing effects of iron oxide nanoparticles (IONPs) and the main approaches to increase their therapeutic efficacy by variable modifications.

## 1. Introduction

Magnetic nanoparticles (MNPs) are nanosized materials (~5 to 150 nm [1,2,3]) with ferro-, ferri-, or supermagnetic properties, which are characterized by an enhanced capability of controlling delivery to target organs using an external magnetic field [4]. Iron oxide nanoparticles (IONPs) are the best known and most used MNPs in biomedicine. Depending on the spatial structure of molecules and the oxidation state of iron, there are three most common forms of IONPs structurally corresponding to iron oxide minerals: magnetite (FeO*Fe_3_O_4_), maghemite (γ–Fe_2_O_3_), and hematite (α–Fe_2_O_3_) [5]. While α-Fe_2_O_3_ nanostructures are applied in designing micro/nanorobots [6,7], FeO*Fe_3_O_4_ and γ-Fe_2_O_3_ are widely used in diagnostics and therapy. In practice, these are employed in the treatment of iron deficiency anemia [8], as a contrast agent for magnetic resonance imaging (MRI) [9,10], and in the development of various antitumor strategies: magnetic hyperthermia [11,12], targeted delivery, and tumor sensitization [13,14,15,16].

It was recently reported that supermagnetic IONPs (also called SPIONs) can have a radiosensitizing effect on tumor tissues, which gives them potential applications in cancer radiation therapy (see Section 3). Moreover, some researchers [14,17,18,19] study the capacity of using an applied magnetic field (AMF) for direct transport of SPIONs, which is possible due to their ultra small size and magnetic single domain [20]. This could potentially solve the unresolved problem of efficient delivery, at least in the case of IONPs [21,22]. At the moment, efficient targeting is provided mainly by improving the nanoparticle modification strategies. It should be noted that the immune status of the organism (studied animal or patient) and the tumor microenvironment play important roles in this case. A number of researchers note that the therapeutic efficiency of systemic exposure with IONPs is based on the immune response leading to infiltration of the tumor with cytotoxic T cells (CD8^+^) [16,23,24]. The question arises of whether immunotoxic and other damaging effects caused by the high reactivity of iron can be effectively localized in target (tumor) tissues. This review will consider the main points of the IONPs pharmacokinetics, modern modification approaches imparting antitumor properties, and the mechanisms by which IONPs sensitize tumor tissues to ionizing radiation.

## 2. General Dynamics of the Cellular Response upon Uptake of IONPs

Numerous mechanistic studies [25,26,27,28] demonstrated that the main pathway of cellular uptake of nanoparticles (up to 150 nm in size) is endocytosis. The latter can be of two types: clathrin- or caveolin-dependent and clathrin-/caveolin-independent [29,30]. The dynamics of endocytosis is determined by the cell type and the physicochemical characteristics of nanoparticles, including the type of protein crown and the degrees of agglomeration, diffusion, and sedimentation [31]. Ultrasmall particles (<10 nm) and cationic nanoparticles with a high charge density can penetrate into the cell through nanopores formed as a result of adhesive interactions with the membrane [30,31,32]. Several studies showed that [33,34,35] iron nanoparticles can be distributed from the bloodstream to various target tissues and organs, but the exact mechanisms of their uptake into target areas are only partially understood. For example, the PI3K/Akt/GSK-3β kinase pathways were found to be important mediators of endothelial cell permeability induced by iron nanoparticles [36]. In particular, in order to overcome the histohematic barriers, IONPs can be modified with various hydrophobic molecules that facilitate penetration through the bilipid membrane by diffusion [37,38,39], and magnetic guidance is proposed to overcome the mucosal intestinal barrier [40].

Due to the engagement of iron ions to participate in electron transfer reactions throughout the most important physiological processes (such as DNA synthesis, mitochondrial respiration, and oxygen transport [41]), IONPs can be much more active than other metal oxide particles [42,43]. Therefore, when developing iron-based nanopreparations, special attention should be paid to minimizing cytotoxic reactions due to iron accumulation in healthy tissues [44]. As a rule, IONPs entering the bloodstream undergo opsonization (adsorption of plasma proteins on the surface of particles) with subsequent recognition and absorption by macrophages of the mononuclear phagocytic system [31,37]. Macrophages of the liver (Kupffer cells), spleen, and circulating blood rapidly absorb opsonized nanoparticles and destroy them intracellularly [45]. Ultrasmall particles comparable in size to globular proteins (~5 nm) are likely to undergo renal clearance [46,47].

## 3. The Main Approaches for IONPs Modification

Nanotherapeutic drugs based on IONPs require to be modified throughout all development stages to achieve the following goals: (i) obtaining a stable structure; (ii) improving the physical and chemical properties of the surface; (iii) conferring a biocompatibility and the desired properties by functionalization with bioactive molecules; and (iv) imparting the affinity to a certain type of cells (tumor, immune). Surface modifications of IONPs can be carried out both in situ (during the synthesis) and ex situ (after the main synthesis) to improve the control of morphology and physicochemical characteristics [48].

**Figure 1:**
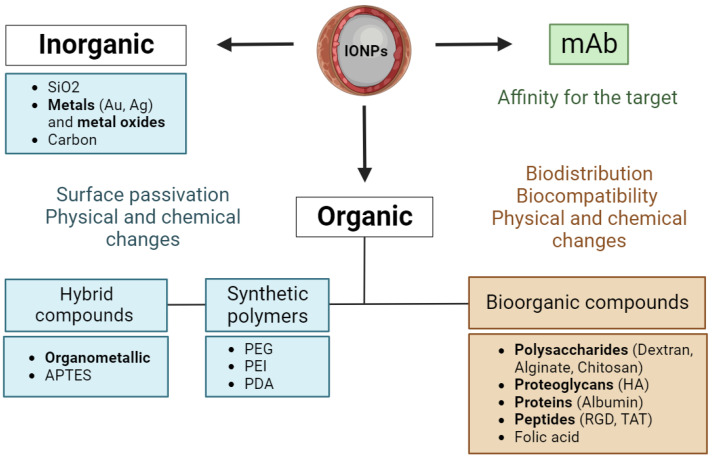
The main approaches to the modification of iron oxide nanoparticles. IONPs—iron oxide nanoparticles, mAb—monoclonal antibodies, and HA—hyaluronic acid.

The primary characteristic of IONPs is their stability. In the absence of any proper surface coating, hydrophobic interactions between iron oxide MNPs cause them to aggregate and oxidize in a physiological environment [49]. It is possible to passivate the surface and improve the physicochemical properties of IONPs with different compounds (Figure 1).

Silicon oxide (SiO_2_) is used to stabilize the surface due to its high thermal and physicochemical stability [50]. Next, gold compounds not only stabilize the core of iron oxide MNPs, but also form magnetoplasmonic nanomaterials with unique surface chemistry and improved magneto-optical characteristics [51,52,53]. Since recently, hybrid carbon coatings [54,55] proved to be a promising nanoplatform catalyst for photodynamic/photothermal therapy. Many synthetic polymers can also improve the stability and pharmacokinetic properties of IONPs. For example, polyethyleneimine (PEI) increases the permeability of IONPs [56,57] and stimulates the production of pro-inflammatory cytokines [56]. Polydopamine (PDA) increases the efficiency of binding various biomolecules for targeted delivery [49,58,59,60], while polyethylene glycol (PEG) improves physical properties, including magnetic ones [61,62,63].

Various bioorganic molecules can be used to impart biocompatibility. For instance, there are polysaccharides as dextran [64,65] and alginate [66]), proteins as albumin [67], and biopolymers that are almost ubiquitous in biological tissues as hyaluronic acid (HA) [68]. Among the common effective strategies is also modification by peptides such as αvβ3 integrin (RGD) [69,70], transactivating transcription activator (TAT) [69,71], and chlorotoxin (CTX) [72]. With regard to the latter, Sophie Laurent et al. presented a detailed review of combinations of IONPs with various peptides [73] and their effects in cellular and animal models.

In addition to the main modifications that ensure stability and biocompatibility, it is possible to functionalize the surface of nanoparticles with monoclonal antibodies to impart affinity to the target [74]. This strategy is often used when targeting a tumor, since a large number of antigens are present on the surface of tumor cells. Typically, there are used tumor-specific antibodies such as anti-HER2 (showed for SK-BR-3, MDA-MB-453 cells and mice [75,76]), anti-MUC1 (showed for MDA-MB-231, MCF-7 cells [77,78]), anti-EGFR for glioblastoma [79,80], anti-VEGF for glioma [81,82], etc. There is also a possibility to combine the approaches. For example, it was demonstrated that in vivo efficacy of dextran iron nanoparticles conjugated with two mAbs was increased against antibodies separately. While the first mAb was targeted to block the signal of the inhibitory PD-L1 checkpoint, the second mAb stimulated T-cells through the costimulatory molecule 4-1BB [83]. Finally, within combined approaches, there is a possibility to increase tumor infiltration with “magnetized” T-cells. Since leukocytes are the first cells that come into contact with intravenously administered nanoparticles, magnetic iron oxide nanoparticles associated with the cell surface can be concentrated within the tumor due to an external magnetic field [84].

## 4. Antitumor Effects of Iron Oxide Nanoparticles

The prospects of the use of iron oxide nanoparticles are driven by its high reactivity, which can be potentially localized within tumor cells using various strategies. Among them are functionalization with pH-dependent groups, modification with specific antibodies, controlled delivery using an applied magnetic field, and synthesis of conjugates with targeted antitumor agents to enhance their effect [13,14,16,74,75,76,77,78,79,80,81,82,85,86,87,88].

Most of the approved antitumor nanodrugs are parenteral-administrated conjugates of nanocarriers (nanoparticles) with small molecular weight chemotherapeutic agents (such as doxorubicin) [89,90]. The targeted action of IONPs as nanoenhancers is based on the fact these can specifically accumulate in the vascularized part of a solid tumor [91,92], exerting immunogenic and damaging effects at different levels [93,94,95,96]. This was demonstrated when studying iron oxide NPs as independent agents in breast cancer models, e.g., MDA-MB231 [97,98], prostate cancer (e.g., PC3, DU145 [99,100]), liver cancer (e.g., HepG2 [101]), brain tumors (e.g., U87 and GL-261 [102,103]), and others. The accumulation of nanoparticles inside the tumor is possible due to the inherent tumor effect of enhanced permeability and retention (EPR). EPR is justified the fact of rapid vascular growth, occurring during tumor development, leads to the formation of defective endothelial architecture and wide pores, which makes it possible to selectively extravasate nanoparticles [104]. In addition, lymphatic outflow is disturbed in the tumors, so the particles, penetrating through the pores, are retained in the tissue [105] and exert their characteristic toxic effects. The EPR was explored in the context of IONPs in various tumor models [106,107,108,109,110,111]. However, Jun Wu reported [112] that interstitial fluid pressure and high density of the tumor tissue make it difficult for the drug to penetrate deep into the tumor. Several significant characteristics of tumors identify whether the EPR may be more significant in solid tumors: (1) substantial tumor neovascularization with blood vessel abnormalities; (2) increased expression of inflammatory factors; and (3) low or loss of drainage in inflammatory systems [112,113].

It was also suggested that the antitumor activity of iron-based nanoparticles is associated with the ferroptosis induction [114,115,116]. The release of ferrous or ferric iron ions in the acidic pH of lysosomes during endocytosis triggers a cycle of Fenton and Haber–Weiss reactions, resulting in the formation of reactive oxygen species. That leads to lipid peroxidation and damage to intracellular macromolecules [117,118].

Ferroptosis reactions are not the only advantage related to magnetic iron oxide nanoparticles. Since SPIONs are ferrofluids whose biodistribution can be controlled by an external magnetic field, this quickly found application as an MRI contrast agent [9,10]. Subsequently, according to the same principle, SPIONs began to be used for magnetic nanothermotherapy. Directed by an applied magnetic field into a tumor, SPIONs generate heat due to fluctuations in magnetic moment [16] and selectively penetrate tumor cells, exposing them to lethal hyperthermia [119,120]. The first evidence for the success of magnetic hyperthermia was presented by Gordon et al. in 1979. They observed histological signs of tumor necrosis in rats with an increase in temperature of 8 °C at an AMF frequency of 450 kHz without side effects or toxic reactions to MNPs [121,122]. At the molecular level, death occurs due to protein denaturation, DNA damage, and activation of various apoptotic pathways [123,124,125]. At the cellular level, heating also increases the release of heat shock proteins into the extracellular environment and increases the functional activity of innate immunity cells: NK killers (through NKG2D activation), macrophages, and dendritic cells [126,127,128]. Dendritic cells (DCs), in turn, take up heat shock protein/tumor antigen complexes, present tumor antigen to T cells and DC migration to lymph nodes, where T cells are activated in an MHC-dependent manner and delivered to tumor cells, passing through venules with high endothelium. Ultimately, activated CD8^+^ T cells attack and cause tumor cell death.

Several authors showed the potency of nanothermotherapy for various in vivo tumor models: liver cancer [129], prostate cancer [130,131,132,133], brain or central nervous system cancers [134,135,136], and melanoma [122,137]. Magnetic hyperthermia is often used in combination with radiation therapy for tumor radiosensitization. This is due to the fact that hyperthermia increases perfusion and oxygenation of hypoxic tumor cells that are resistant to ionizing radiation. In addition, hyperthermia acts mainly at acidic pH and in the radioresistant S-phase of the cell cycle. This means that radiation therapy and hyperthermia complement each other; as a result of radiation therapy, formed free radicals damage the DNA of tumor cells, while hyperthermia inhibits its repair [138,139]. Despite a growing body of basic research and encouraging results both in vitro and in vivo, there are the technical difficulties of developing magnetic field applicators. Difficulty of maintaining frequencies and field characteristics suitable for clinical use, while adhering to the safety rules, is the main obstacle that holds the development of this method to clinical application [140].

Thus, the prospects for the use of magnetic iron oxide nanoparticles are due to the potential of their application within the framework of multifunctional technologies. This includes magnetic IONPs associated with a cytostatic drug (acting as its carrier) and a means to control how it can be moved around the human body (a source of an external magnetic field or implants placed into the body). Using the IONPs’ ability to local heating can significantly increase the effectiveness of treatment by providing thermal destruction of the tumor. It is also possible to control the dosing of drugs through the use of a shell that has the desired properties in terms of its degradation, while ensuring a controlled release of the cytostatic.

## 5. Mechanisms of Radiosensitization by Iron Oxide Nanoparticles

Since the appearance of data about the potential antitumor IONPs’ activity for glioblastoma, prostate, lung, liver, and breast cancers [97,98,99,100,101,102,103], a number of studies consider the combination of IONPs with ionizing radiation (IR) as a promising method of tumor treatment [141,142,143,144,145]. It is known that radiosensitizing effects quantitatively depend on the type of IR, as well as on the characteristics of nanoparticles, such as shape, size, surface coverage, and concentration [146]. We collected the data about some synthesized IONPs with proven radiosensitizing potency (Table 1).

**Figure 2:**
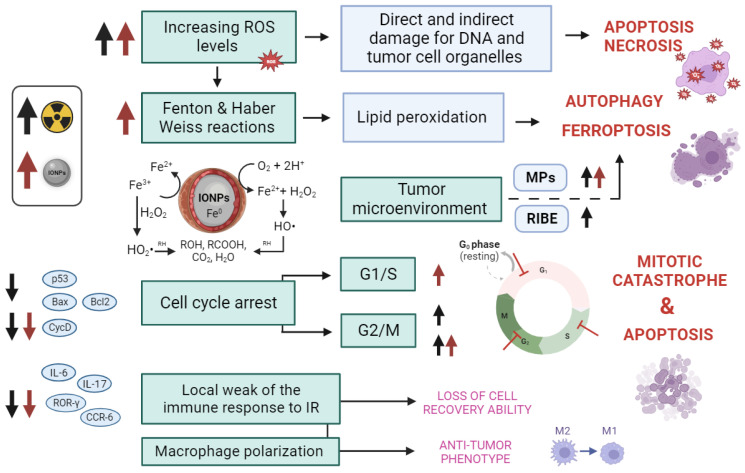
General mechanisms of radiosensitization by iron oxide nanoparticles. A black arrow corresponds to ionizing radiation action, and a red arrow corresponds to IONPs action. The arrows correspond to the ways of radiosensitivity enhancement. IR—ionizing radiation, MPs—microparticles, RIBE—radiation-induced bystander effect, and ROS—reactive oxygen spp.

We decided to summarize the mechanisms by which IONPs can sensitize a tumor to IR and at the cellular and molecular levels (Figure 2), and establish the origin of each one.

### 5.1. Increasing the Traumatic for Tumor Cells ROS Levels

Reactive oxygen species (ROS) are an integral part of a normal aerobic metabolism, this includes H_2_O_2_ and all highly reactive unstable metabolites of molecular oxygen (O_2_^−^, HO^−^, and HO_2_^−^). Whereas biological tissues interact with IR, ROS number increases sharply, and the balance of redox processes is disturbed [156]. Iron oxide nanoparticles enhance this effect: the dissolution of O_2_^−^ from metal oxide can saturate the cell with oxygen and promote the formation of ROS, as well as overcome hypoxic resistance to radiation therapy. In addition, any dissolved metal ions can act as oxidizing and reducing agents and increase the ROS production, for example, during the Haber–Weiss reactions and the Fenton cycle [157,158]. All of these can provoke mitochondrial dysfunction, high autophagic activity, and ultimately, cell death, e.g., it was demonstrated in PC12 rat pheochromocytoma cells [159]. This effect can be localized in tumor cells in several ways. First, due to the previously mentioned effect of increased permeability and retention, the particles are predominantly localized in tumors. This increases the invasiveness of tumor cells, and due to the small size of SPIONs, the absorption effect is much higher. Secondly, in order to ensure the selectivity of this effect for tumor cells, the modifications as pH-dependent peptides (based on the Warburg effect [160,161]), monoclonal antibodies specific to tumor antigens [77,79,80,81,82,162,163,164] are used. Thirdly, within the framework of the de novo approach, it is possible to use the guidance of SPIONs into the tumor using an applied magnetic field, which will enhance the effect in combination with any of the described strategies [14,165].

Thus, IONPs can enhance the radiobiological response either by increasing the accumulation and activity of free radicals and the corresponding general cytotoxicity, or by controlled delivery of particles that are highly tropic for tumor cells and exhibit selective cytotoxicity.

### 5.2. Ferroptosis as a Special Case of Antioxidant Deficiency

Ferroptosis is an iron-dependent, oxidation-regulated cell death characterized by the accumulation of peroxide lipids within the cell. It makes sense that ferroptosis is associated with insufficiency of antioxidant defense systems [166], in particular, the glutathione-enzyme autonomous complex. Numerous studies confirmed that iron oxide nanoparticles induce ferroptosis [118,167,168,169,170], which is quite expected due to disturbance of iron homeostasis and activation of Fenton cycles [171]. It was recently demonstrated that tumor cells can be hypersensitive to radiation due to increased ferroptosis, which also correlates with better response and increased survival in cancer patients with radiotherapy [172,173,174].

Ionizing radiation itself can enhance ferroptosis through parallel mechanisms:

- By increasing the expression of acyl-CoA synthetases 4 (ACSL4) with the formation of oxidized polyunsaturated fatty acyl fragments (PUFA-PL) in membrane phospholipids [173,175,176,177];
- By DNA damage, resulting in the activation of ATM, which inhibits the production of SLC7A11, a key component of the cystine/glutamate transporter. It can permanently deplete glutathione (GSH) and inhibit glutathione peroxidase 4 (GPX4). Further, it weakens the defense system against ferroptosis mediated by the SLC7A11-GSH-GPX4 signaling pathway and disrupting redox homeostasis [178,179];
- By DNA damage that also increases the expression of TFR1 in cells with mutations in the RAS gene, as well as decreases the expression of iron-storing ferritin, which leads to an increase in Fe^2+^ content in the cell [178,179,180,181];
- By DNA damage, causing the cyclic GMP-AMP synthase (cGAS) signal of the DNA sensor to activate the cGAS-STING1 pathway, resulting in autophagic-dependent ferroptosis via lipid peroxidation [182];
- By promoting the release of microparticles (MPs) from tumor cells, which alter the tumor microenvironment, enhance the antitumor effect, and mediate radiation-induced bystander effects (RIBE) in tumor cells, essentially causing ferroptosis [183].

The researchers suppose that ionizing radiation used in conjunction with ferroptosis inducers (in our case, iron oxide nanoparticles), is promising for further research in cancer therapy, since the potency of radiation therapy can be greatly increased [172].

### 5.3. Cell Cycle Arrest

The ability to recognize and repair DNA breaks is one of the criteria for determining radiosensitivity at the cellular level [184]. Most IR-induced double-strand breaks (DSBs) are repaired by nonhomologous end joining. However, a subset of IR-induced DSB in the S and G2 cell cycle phases can be repaired by homologous recombination using sister chromatids as a repair template [185]. This mediates repair of damaged replication forks in the S phase and promotes radioresistance in the S and early G2 phases [186]. Dividing cells in the late G2 and M phases are not capable of repairing DNA double-strand breaks [187], which makes them radiosensitive. Impairment of the transition from dormancy to mitogenesis, i.e., de facto independence from the G1 checkpoint, suggests a greater tumor dependence on the intra-S-phase and G2/M checkpoints for the restoration of radiation damage. Therefore, targeting these checkpoints can selectively sensitize tumor cells to IR [188,189,190]. Thus, normal cells can still stop at the G1 checkpoint and repair damage, while tumor cells, skipping through G1, enter mitosis and die as a result of a mitotic catastrophe, not having enough time to recover [191].

IONPs themselves can affect different phases of the cell cycle. For example, ultrasmall Fe_3_O_4_ NPs notably inhibited DNA synthesis and enhanced cell apoptosis by inducing S-phase arrest, and in this way reduced the MCF-7 cell growth and proliferation [192]. It disturbed the mRNA expressions of HMOX-1, GCLC, and GCLM, inducing the high ROS production and decreased GSH. That led to a serious oxidative damage and growth inhibition for MCF-7 cells. In another study, treatment with Fe_3_O_4_ nanoparticles caused cell cycle arrest at the G2/M phase in PC12 cells, which was accompanied by increased expression of the P53 gene without affecting the downstream P21 and GADD45 signaling pathways [193].

The joint use of IONPs and IR showed good results in several studies. For example, the viability of U87 cells was significantly reduced after treatment with X-rays and iron oxide nanoparticles (Fe_3_O_4_@APTS) compared with treatment with X-rays alone [194]. In addition, the percentage of cells in the G2/M phase and the percentage of apoptotic cells were significantly higher in the Fe_3_O_4_@APTS irradiated group than in the X-ray-only group (*p* < 0.05). Popescu R.C. et al. demonstrated that preliminary exposure to ionizing radiation on MG-63 human osteosarcoma cells promoted enhanced internalization of doxorubicin-conjugated nanoparticles (NP-DOX) [195]. This was accompanied by premature entry of MG-63 cells into the G2/M phase. At 48 h after treatment, cells re-entered G1 (similar to untreated cells) and then underwent mitotic catastrophe. At the same time, the NP-DOX particles themselves showed hemocompatibility, the absence of systemic cytotoxicity, and did not cause histopathological changes.

Thus, iron oxide nanoparticles can increase the radiosensitivity of the tumor by affecting the cell cycle, i.e., either stopping it in the most radiosensitive G2/M phase, or stopping replication and programming a proportion of radioresistant S-phase cells for death. All this can lead not only to increased radiosensitization, but also to sensitization to antitumor therapeutic agents [76], which can be used to load IONPs.

### 5.4. Local Weakening of the Immune Response to Radiation

As already mentioned, the immune response to IONPs depends on the individual characteristics of the organism (immune status), so the clinical implementation is often delayed or even stopped due to concomitant immunotoxicity [196]. One of the variants of the immune response to iron oxide nanoparticles is local immunosuppression, upon which the anti-inflammatory action of cytokines is inhibited. This prevents cells from efficiently recovering from ionization stress and thus potentially has a radiosensitizing effect.

There is evidence that immune responses mediated by Th1 and Th2 T helpers are suppressed by IONPs in OVA-sensitized mouse models [197,198]. As a result, a decrease in the expression of IL-6, IL-17, ROR-γ, and CCR-6 [198] was shown; teamwise, these results indicate that T cells are a sensitive target in the immune system to IONPs. It was also recently found that AntiPD-L1 antibody-conjugated AuNP@SPIOs are able to polarize tumor-associated macrophages (TAMs) from M2-like (pro-tumor) to M1-like (anti-tumor) type, which is critical for the effect of radiotherapy [199]. The local radiosensitizing effect of iron oxide nanoparticles may also result from reprogramming (weakening) of the immunoreactive microenvironment [200,201].

### 5.5. Other Possible Mechanisms

Among the insufficiently studied possible mechanisms of radiosensitization, one can also assume the effect of iron oxide nanoparticles on the chromatin structure. It is known that actively transcribed genes are surrounded by large-scale domains of radiosensitive chromatin, and that replicating DNA with an open chromatin structure is more sensitive to DSB induction by IR [202]. In the process of detecting and repairing DNA damage, chromatin must locally open the structure so that repair mechanisms have access to the primary DNA sequence and can repair effectively. Recent studies [203] showed that IONPs reduce hepatocyte chromatin homogeneity in a dose-dependent manner. However, whether this is a cause or a consequence of cell death remains to be established as the detected changes could be discrete morphological changes in chromatin distribution occurring at very early stages of programmed cell death. Nevertheless, this effect requires further study, and it should be considered as a possible synergistic mechanism of radiosensitization, since currently, there are radiosensitizers that affect the structure of chromatin (among them are vorinostat, belinostat, and panobinostat [204]).

## 6. Future Prospects

Iron oxide NPs, especially supermagnetic ones, have great prospects for use in the combined chemoradiotherapy of cancer. Firstly, extra-small size makes IONPs potentially penetrable through histohematic barriers. Thus, when properly modified, they can have a powerful cytostatic/cytotoxic effect on tumor cells. Secondly, due to supermagnetism, they are able to be distributed with an applied magnetic field, the question remains only in the technologies used and their release into wide medical practice. Thirdly, due to hyperthermic effects, IONPs can locally heat the cell during irradiation, which potentially increases the effectiveness of one procedure (with prospect to reduce the course length). At that moment, there is a problem of insufficient knowledge about the signaling pathways through which IONPs affect the cell conditioning radiosensitivity enhancement. Studying of the signaling pathways will make it possible to better predict the effectiveness of nanoparticles along with its surface activity. Since modified with various peptide complexes, monoclonal antibodies, and other molecules, IONPs can mediate downstream pathways in different manner. One more direction remains open for development: the combined therapy with an applied magnetic field. It is still necessary to understand how AMF can be applied during oncotherapy course. In order to take full advantage of IONPs in the development of an effective multi-targeted radiosensitizing drug, all of the above must be taken into account.

## 7. Conclusions

The pharmacodynamic activity of iron oxide nanoparticles is conditioned by its high reactivity. Thus, despite the obvious advantage of magnetic properties, giving the ability to localize IONPs in the tumor, the design of each iron nanopreparation should be thought out as carefully as possible. Surface modifications affect all the main nanodrug parameters, such as physicochemical properties of IONPs, affinity to the target, biocompatibility, and safety of the whole. Modern modification strategies make it possible to create an effective theranostic agent that can both provide an independent therapeutic effect and sensitize additive anticancer therapy. We collected and summarized data on possible mechanisms contributing to the increase in tumor sensitivity to IR using iron oxide nanoparticles.

The sensitization of radiation therapy with IONPs is mediated, at first, by an increase in the number of free radicals due to an increase in the localized emission of secondary electrons from the nanoparticles. Moreover, the lower pH of the medium correlates with enhanced stimulation of ROS release by IONPs, thus the Warburg effect is the probable reason for why IONPs work better in tumor cells. Electron transfer reactions somehow affect all cellular processes, contributing to extensive damage to cellular organelles, mitochondrial stress, and initiation of the protein and lipid oxidation, which ultimately leads to a significant percentage of death by the ferroptosis mechanism. Speaking about the mechanisms of radiosensitization by iron oxide nanoparticles, we cannot overlook the immunoreactivity of iron complexes. Depending on the immune status, IONPs can sensitize the tumor to radiation therapy by reprogramming the immunoreactive cellular microenvironment. It can manifest in the direct weakening of T1-helper and Th2-cell immunity by reducing the expression of IL-6, IL-17, ROR-γ, and CCR-6. It is also worth mentioning the influence of the generally recognized effect of increased permeability and retention (EPR) of iron oxide nanoparticles by tumor cells. Due to EPR, any sensitizing effects are noted to a greater extent in tumors, at least because of the fact that tumor cells absorb many times more nanoparticles than healthy epithelial ones. Along with EPR, we can use the principle of magnetic hyperthermia for IONPs, which will absolutely provide an increased delivery efficiency and a corresponding enhanced targeted action, unlike the same EPR. Finally, iron oxide nanoparticles can promote radiosensitivity by influencing the cell cycle either by stopping it in the most radiosensitive G2/M phase or by stopping replication and programming a proportion of radioresistant S-phase cells to die. In this case, IR and IONPs enhance each other in damage effects within the phase, continuing to subject cells to oxidative stress and to provoke ferroptosis.

Thus, our review emphasizes the relevance and prospects for research IONPs in cancer therapy and to improve the understanding the mechanisms of IONPs radiosensitization for expanding the possibilities of their therapeutic use.

## Figures and Tables

**Table 1:** Iron oxide nanoparticles with radiosensitizing potency.

Nanoparticles	Modification	Size	Radiosensitization Scope	Reference
IONP	Gold coating	55 nm	Melanoma	[147]
Iron oxide	Dextran-coated with conjugated TAT-peptide	127 nm	A459 carcinoma cells	[141]
Commercially available iron oxide (Plain-NanoMag)	Dextran-coated	20 nm	Human prostate carcinoma cell	[142]
SPION	Dextran-coated	6 nm	Human glioblastoma	[148]
Iron oxide	Dextran-coated	15 nm	MCF7 and HeLa cells	[149]
SPION	Poly (ethylene glycol) methyl ether coating	9 nm	Melanoma	[150]
Iron oxide	Silica-coated	164 nm	MCF7 human breast cancer cells	[151]
SPION	Sodium citrate coating	6–25 nm	MCF-7 (human breast adenocarcinoma), MDAMB-231 (human mammary gland carcinoma) and MDAH-2774 (human ovarian carcinoma) cell lines	[152]
SPION	(3-Aminopropyle)-Triethoxysilane	166 nm	HPV-negative (HPV-) HNSCC cell lines	[153]
Fe_3_O_4_-Au NPs	TPP-PEG4 (to mHsp70)	4 nm	TNBC 4T1 и MDA-MB-231 (Triple-negative breast cancer)	[154]
cetuximab-IONPs	Ceturimab (mAb to EGFR)	10 nm	Glioblastoma (U87MG, mice)	[155]

## Data Availability

Data sharing not applicable. No new data were created in this study. Data sharing is not applicable to this article.
